# Analysis of the transcriptome of the Indonesian coelacanth *Latimeria menadoensis*

**DOI:** 10.1186/1471-2164-14-538

**Published:** 2013-08-08

**Authors:** Alberto Pallavicini, Adriana Canapa, Marco Barucca, Jessica Alfőldi, Maria Assunta Biscotti, Francesco Buonocore, Gianluca De Moro, Federica Di Palma, Anna Maria Fausto, Mariko Forconi, Marco Gerdol, Daisy Monica Makapedua, Jason Turner-Meier, Ettore Olmo, Giuseppe Scapigliati

**Affiliations:** 1 Dipartimento di Scienze della Vita, Università di Trieste, Trieste, Italy; 2 Dipartimento di Scienze della Vita e dell’Ambiente, Università Politecnica delle Marche, Ancona, Italy; 3 Dipartimento per l’Innovazione nei Sistemi Biologici, Agroalimentari e Forestali, Università della Tuscia, Viterbo, Italy; 4 Faculty of Fisheries and Marine Science, University of Sam Ratulangi, Manado, Indonesia; 5 Broad Institute of MIT and Harvard, Cambridge, MA, USA

**Keywords:** Coelacanth, *Latimeria menadoensis*, Transcriptome, *de novo* assembly, RNA-seq

## Abstract

**Background:**

*Latimeria menadoensis* is a coelacanth species first identified in 1997 in Indonesia, at 10,000 Km of distance from its African congener. To date, only six specimens have been caught and just a very limited molecular data is available. In the present work we describe the *de novo* transcriptome assembly obtained from liver and testis samples collected from the fifth specimen ever caught of this species.

**Results:**

The deep RNA sequencing performed with Illumina technologies generated 145,435,156 paired-end reads, accounting for ~14 GB of sequence data, which were *de novo* assembled using a Trinity/CLC combined strategy. The assembly output was processed and filtered producing a set of 66,308 contigs, whose quality was thoroughly assessed. The comparison with the recently sequenced genome of the African congener *Latimeria chalumnae* and with the available genomic resources of other vertebrates revealed a good reconstruction of full length transcripts and a high coverage of the predicted full coelacanth transcriptome.

The RNA-seq analysis revealed remarkable differences in the expression profiles between the two tissues, allowing the identification of liver- and testis-specific transcripts which may play a fundamental role in important biological processes carried out by these two organs.

**Conclusion:**

Given the high genomic affinity between the two coelacanth species, the here described *de novo* transcriptome assembly can be considered a valuable support tool for the improvement of gene prediction within the genome of *L. chalumnae* and a valuable resource for investigation of many aspects of tetrapod evolution.

## Background

One of the most important transitions in vertebrate evolution was the arising of terrestrial vertebrates, which entailed considerable morphological changes related to the acquisition of novel functions by pre-existing and, in several cases, pre-adapted structures, like the evolution of lobe fins into tetrapod limbs. The terrestrial vertebrates would have derived from fossil forms of lobe-finned fishes, a highly successful group in the Devonian (400 Mya), with hundred species populating the Gondwana supercontinent’s oceans and river systems [1,2].

Until 1938 only two sarcopterygian taxa were considered to have survived post-Devonian extinction: the dipnoi (lungfish), with three extant genera, and the tetrapods, with ~23,500 species. Hence the clamour when the first living coelacanth (*Latimeria chalumnae*), a fish considered extinct, was found off the estuary of river Chalumna, in South Africa [3,4]. In 1997 a specimen of another *Latimeria* population was identified by Mark V. Erdmann in a fish market in Manado Tua (Sulawesi, Indonesia) [5]. The distance between the two sites (more than 10,000 Km) and the early molecular findings [6,7] led to the identification of the latter specimen as belonging to a distinct species, called *L. menadoensis*. Several individuals of *L*. *chalumnae* have been fished to date, as opposed to only six individuals of *L. menadoensis*. Specimens of Indonesian coelacanth are therefore very rare and constitute a valuable scientific resource and a mine of precious genetic information.

The main molecular and morphological studies of the genus *Latimeria* have addressed the evolutionary relationships linking lungfishes, coelacanths and tetrapods. Their results have however been discordant, since different datasets have sustained different hypotheses [6-20]. *L. menadoensis* has also been the subject of other molecular investigations aimed at characterizing some genes of evolutionary interest: *Hox* genes [21-23], *ParaHox* genes [24], the *Protocadherine* cluster [25], the *RAG1* and *RAG2* genes [11], *Sonic hedgehog* gene and its enhancers [26], visual pigments [27], a *Heat Shock Protein 70*[28], neurohypophysial hormones [29], and vitellogenins [30]. Furthermore some transposable elements (TEs) were characterized in this species [31-34]. The importance of *L. menadoensis* has led to the study of its whole mitochondrial genome [10,35] as well as to partial sequencing of a BAC library, which has made ~5 Mb of genomic sequences available to databases [36]. Furthermore, correlations between quantitative and compositional characteristics of the genome of *L. menadoensis* were considered in Makapedua et al. [37].

Over the last few years next generation sequencing technologies (NGS) have revolutionized the fields of genomics and transcriptomics, providing the opportunity to analyze genomes and transcriptomes with high sequencing depth in a relatively short time in comparison with Sanger sequencing. The molecular data obtained with such technologies, applied to a rising number of organisms, are proving steadily important to study their relationships at the macro- and micro-evolutionary levels. In this respect, having in mind that genes are targets of evolution-driven changes that lead to the different morphology of animals, in the framework of studies of genomic features of coelacanths [38], we examined the *L. menadoensis* transcriptomes of liver and testis using deep-sequencing techniques.

The liver is one of the most suitable tissues in that it participates more than other organs in a range of physiological processes and contains cell types endowed with distinct roles and functions. Considering the different sex determination mechanisms occurring across vertebrates, the expression in testis is interesting to better understand the genes involved in these processes and their evolution since the systematic position of *Latimeria*. Furthermore, this tissue proved to be particularly suitable and useful for deep RNA-seq, as it expresses a broad range of different transcripts, permitting the assembly of a high number of full length sequences. In fact, about a half of the sequences included in the high quality set of 66,308 contigs was estimated to have been correctly assembled to the full length.

The *de novo* transcriptome assembly was able to significantly enhance the global view of the sequences expressed in coelacanth, overcoming the limitations linked to the automated and conservative, protein coding gene-focused, prediction by Ensembl, and providing a remarkable amount of information concerning expressed sequences produced by non-annotated genes. This knowledge provided significant information not only to investigate important biological processes and metabolic pathways in *Latimeria*, but also to acquire information on the origin of tetrapods and on the possible evolutionary dynamics relative to the genes involved in the transition from aquatic to terrestrial vertebrates.

## Results

### *Latimeria menadoensis* transcriptome sequencing

The Illumina sequencing procedure generated 145,435,156 raw nucleotide paired-end reads (76,932,818 and 68,502,338 reads from liver and testis, respectively). The average read length for liver was 97.28 bp, corresponding to a complete dataset of 7.48 GB of sequence data, while the deep RNA-seq of testis produced reads slightly shorter, with an average length of 96.22 bp, accounting to 6.59 GB of sequence data. Following the processing steps involving the trimming of adapters and low quality bases, and the removal of short reads and of reads originated by ribosomal RNA, the two sequence sets were significantly reduced to 47,470,578 and 41,401,836 high quality sequencing reads from liver and testis, respectively. Therefore a total of 88,872,414 reads were used for the *de novo* assembly. A summary of the trimming step statistics is reported in Table 1. A detailed report of quality and statistics for the reads used for the *de novo* transcriptome assembly is presented in Additional file 1.

**Table 1:** Trimming report

	**Liver**	**Testis**
Number of reads before trimming	76,932,818	68,502,338
Reads kept after trimming	64,099,318	55,326,118
Percentage of discared reads	10.96%	13.69%
Reads average length before trimming	97.28	96.22
Reads median length before trimming	104	104
Reads average length after trimming	103.4	102.9
Reads median length after trimming	104	104
Ribosomal RNA reads	16,628,740	13,924,282
Percentage of ribosomal RNA reads	21.61%	20.33%
Number of high quality reads	47,470,578	41,401,836

### *De novo* assembly

The *de novo* transcriptome assembly performed with Trinity [39] by using both liver and testis reads generated a total of 306,882 contigs. The filtering step used to select only the longest transcript per gene produced 223,365 contigs, and the additional step applied to remove redundant sequences by MIRA 3.4.0 [40] and to filter sequences shorter than 250 bp further reduced the Trinity assembly to a set of 105,653 transcripts. The *de novo* assembly produced with the CLC Genomic Workbench 4.5.1 (CLC Bio, Katrinebjerg, Denmark) generated 149,339 raw contigs. The high quality subset of protein-coding sequences selected to integrate the Trinity assembly, as described in the methods section, comprised 48,846 sequences. A total of 8,496 CLC contigs were detected by BLASTn as matching existing Trinity contigs and significantly longer than them. The corresponding Trinity contigs were therefore replaced. The remaining 40,350 CLC contigs were discarded, as they could not significantly improve the Trinity assembly.

**Figure 1:**
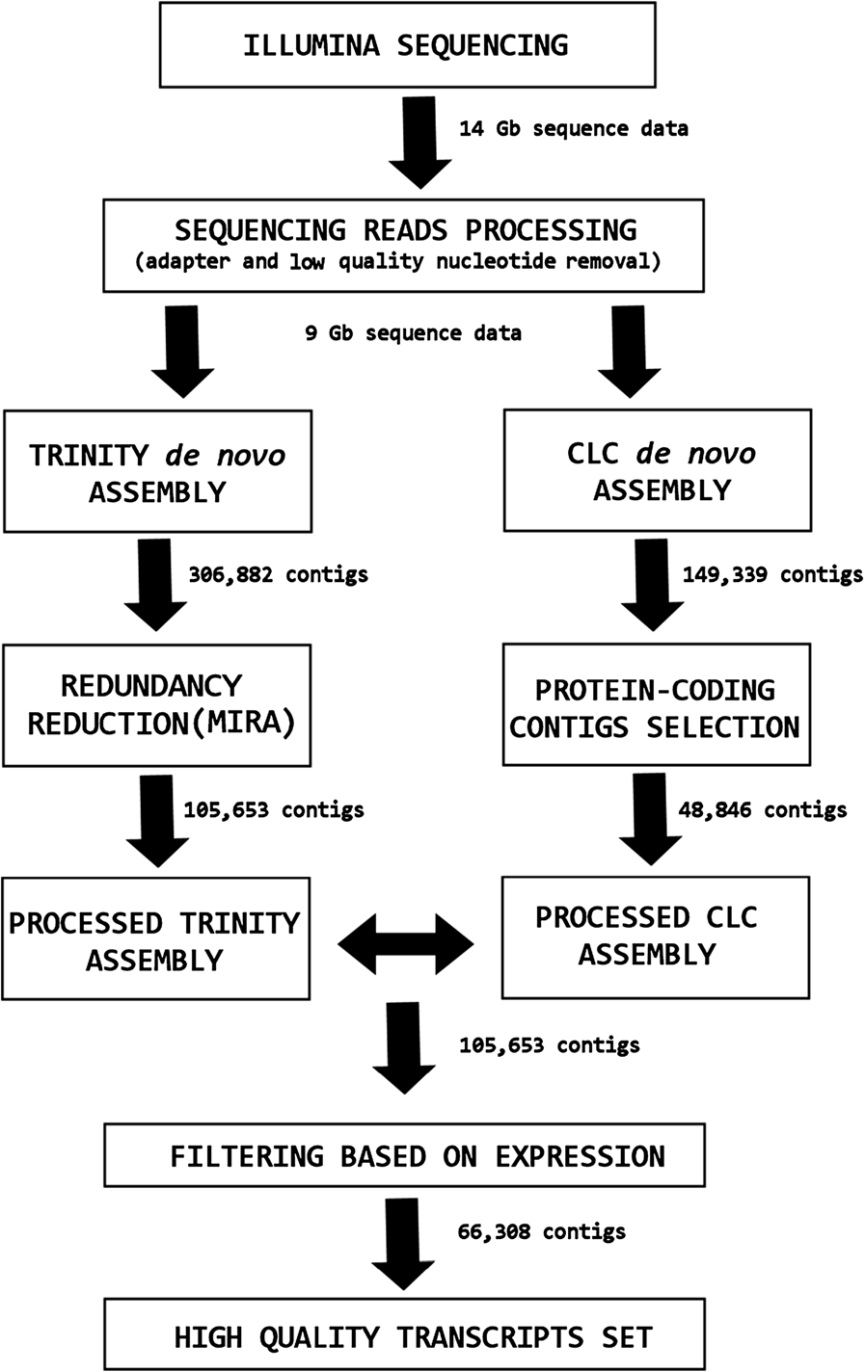
**Graphic summary of the combined *de novo *assembly strategy and filtering steps applied to generate the final high quality transcripts set comprising 66,308 sequences.**

A total of 105,653 contigs was obtained following the combination of the data generated by the two *de novo* assemblers. Finally, the filtering step applied to remove poorly covered sequences, resulting from the fragmentation of transcripts expressed at particularly low levels, reduced the contig number to a final high quality set of 66,308 sequences. A detailed graphical summary of the strategy used and of the results obtained by the *de novo* assembly of *L. menadoensis* transcriptome is shown in Figure 1.

### Assembly quality assessment

**Figure 2:**
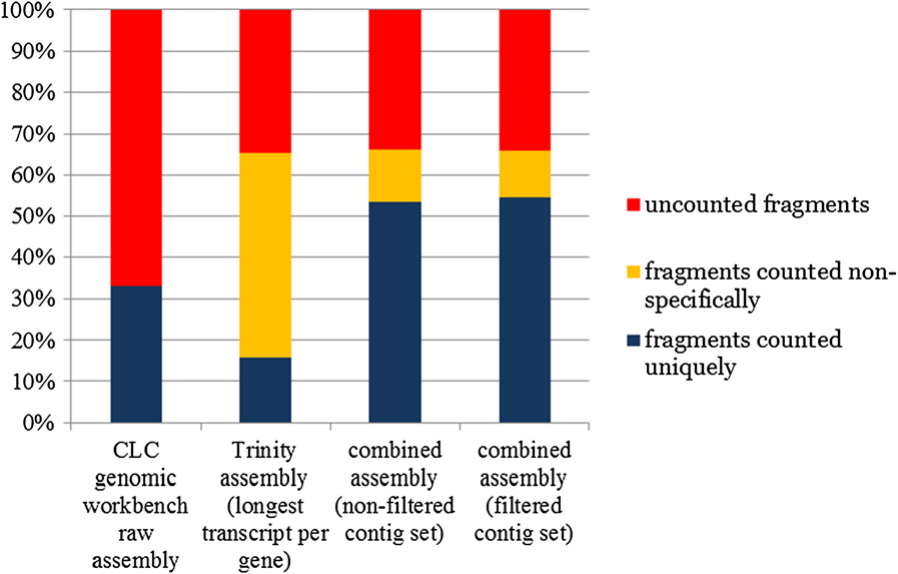
**Sequencing read usage in the assemblies and assembly redundancy estimated by RNA-seq mapping.** Redundancy is calculated as the number of fragments mapping non-specifically on multiple contigs. Fragments mapping on contigs as broken read pairs were not counted.

**Figure 3:**
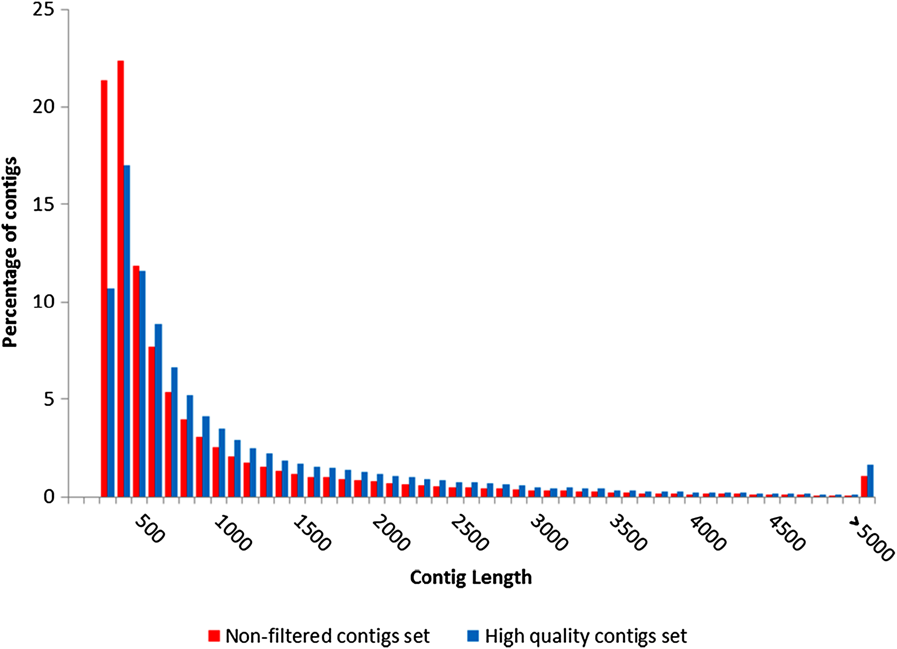
**Comparison of contig length distribution before (red) and after (blue) the filtering step based on average sequence coverage.** The reduction of the fraction of short contigs is represented by the shift of distribution towards the right side of the graph. x-axis: length categories, organized in 100 bp intervals. y-axis: percentage of contigs observed per length category.

The goal of these assembly processing steps was to reduce redundancy without losing any valuable sequence data (Figure 2). Despite making use of a large fraction of the original sequencing reads (65.41% of the intact sequence pairs -fragments- could be mapped to the contigs), the raw Trinity assembly was largely redundant, as the mapping of the reads on the assembled contigs revealed 75% of non-specific matches. On the contrary the raw CLC assembly showed virtually no redundancy (~0.01%) but only 33% of sequenced fragments were used to produce the assembly. The sequence redundancy was drastically reduced to 19.21% after the removal of Trinity redundant contigs by MIRA with no loss of sequence data, as the total number of reads mapped on the updated assembly slightly increased (+1.19%) due to the elongation of 8,496 Trinity contigs by CLC. Although a large portion of contigs with low expression was discarded (39,342, accounting for 37.24% out of the total), this did not significantly affect the total number of mapped reads (which only decreased by 0.34%) and contributed to a further reduction of sequence redundancy (which dropped to 17.39%). The comparison between sequence length categories based on average coverage, before and after the contig filtering step (Figure 3), revealed that this procedure was able to sensibly reduce the amount of short sequences, especially those shorter than 500 bp, moving the distribution of contig length towards longer and more reliable sequences.

Transcript fragmentation was assessed with the Ortholog Hit Ratio method [41], which relies on the comparison between the observed length of contigs and the full length of known ortholog sequences of other species, detected by BLASTx. This method is strongly influenced by inter-species divergence and by the different substitution rates observed among genes and can often lead to an under-estimation of transcript integrity [42]. To overcome this imperfection of the method we applied a correction considering in the analysis only highly conserved genes (characterized by a BLAST identity higher than 90%, independently from the hit length). By these means, a sufficiently large set of sequences was analyzed (6,024 contigs), permitting to obtain a reliable estimate of fragmentation within the high quality liver and testis transcripts. The comparison with ortholog sequences revealed that about a half of the contigs were assembled to their full length (Additional file 1: Figure S1e). The mean and median ratios resulted to be 0.72 and 0.86, respectively. Approximately a quarter of the high quality transcript set is expected to be composed by highly fragmented contigs (covering less than 50% of the expected length).

The average length of the contigs obtained, ranging from 250 (the minimum length allowed) to 20,815 bp, was 1,080 bp. The N50 statistic of the assembly (defined as the length of the smallest contig in the set that contains the fewest and largest contigs whose combined length represents at least 50% of the assembly [43]) was 1,761 and 1,081 contigs longer than 5 Kb were obtained (80 contigs were longer than 10 Kb). A summary of the final assembly statistics is shown in Table 2.

**Table 2:** Assembly statistics

Total number of high quality assembled reads	88,872,414
Number of created contigs	66,308
Number of bases in contigs	71,621,287
Average length (bp)	1,080
Median length (bp)	626
N50 (see the text for definition)	1,761
N80 (see the text for definition)	662
N90 (see the text for definition)	438
Longest contig (bp)	20,815
Number of contigs > 5 Kb	1,081
Number of contigs > 10 Kb	80

### Transcript annotation

**Figure 4:**
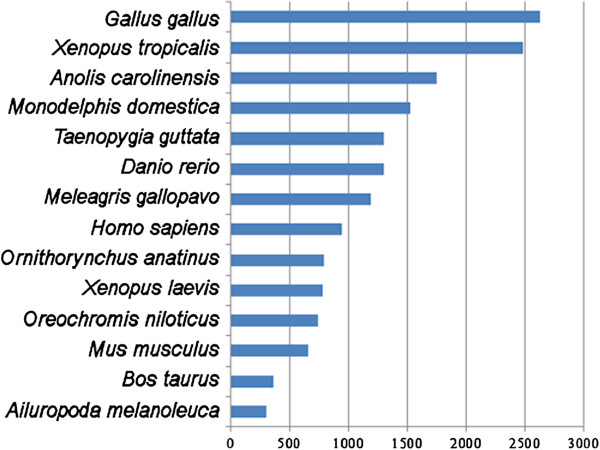
**Top BLAST hit species distribution, obtained by BLASTx against the NCBI non-redundant (nr) protein database.** The number of top BLAST hits per species is shown on the x-axis. Only the 15 most represented species are shown. The complete number of top hits of other organisms is 7,572.

The annotation performed with BLASTx to the NCBI non-redundant (nr) protein database revealed that 23,564 of the assembled contigs (35.54%) had at least one positive hit. 42,744 contigs did not give any BLAST hit by the cutoff of 1x10^-6^. The BLAST top hit species distribution is shown in Figure 4. The BLAST2GO annotation, directly performed on the high quality set of transcripts translated into the six possible reading frames, revealed 42,667 out of 66,308 total sequences bearing at least one InterPro domain, accounting for 64.35% of annotated transcripts. The list of the 25 most abundant InterPro domains is displayed in Table 3, with IPR000719 (Protein kinase, catalytic domain) being the most represented one, with 2,041 annotated transcripts, followed by IPR007087 (Zinc finger, C2H2) and IPR002290 (Serine/threonine-/dual-specificity protein kinase, catalytic domain). The assembled sequences were also annotated with Gene Ontology (GO) terms as described in the materials and methods section, according to the three major GO categories: Cell Component, Molecular Function, and Biological Process. A total of 28,502 transcripts (42.98%) were associated with at least one GO term; concerning the second level of ontology, 6,698 were assigned to a Cell Component category, 13,061 to a Molecular Function category, and 13,030 to a Biological Process category. The summary of Gene Ontology mappings is reported in Additional file 1: Figure S1f. Concerning the cellular localization, the majority of annotated transcripts was assigned to cell (GO:0005623), followed by organelle (GO:0043226) and macromolecular complex (GO:0032991). The largely predominant molecular functions resulted to be binding (GO:0005488) and catalytic activity (GO:0003824). Finally, concerning biological processes, cellular process (GO:0009987) and metabolic process (GO:0008152) were the two GO terms most represented.

**Table 3:** The 25 most abundant InterPro domains revealed by the InterProScan annotation of the high quality coelacanth transcript set

**InterPro domain**	**Description**	**Number of detected contigs**
IPR000719	Protein kinase, catalytic domain	2,041
IPR007087	Zinc finger, C2H2	1,778
IPR002290	Serine/threonine-/dual-specificity protein kinase, catalytic domain	1,472
IPR013783	Immunoglobulin-like fold	1,130
IPR015880	Zinc finger, C2H2-like	1,056
IPR020635	Tyrosine-protein kinase, catalytic domain	981
IPR011009	Protein kinase-like domain	946
IPR020683	Ankyrin repeat-containing domain	927
IPR001680	WD40 repeat	845
IPR001849	Pleckstrin homology domain	834
IPR003961	Fibronectin, type III	822
IPR001452	Src homology-3 domain	792
IPR008271	Serine/threonine-protein kinase, active site	696
IPR001841	Zinc finger, RING-type	687
IPR007110	Immunoglobulin-like	684
IPR017986	WD40-repeat-containing domain	675
IPR020849	Small GTPase superfamily, Ras type	674
IPR013087	Zinc finger C2H2-type/integrase DNA-binding domain	662
IPR000504	RNA recognition motif domain	661
IPR002110	Ankyrin repeat	659
IPR013083	Zinc finger, RING/FYVE/PHD-type	614
IPR001478	PDZ domain	600
IPR015943	WD40/YVTN repeat-like-containing domain	600
IPR001650	Helicase, C-terminal	575
IPR016024	Armadillo-type fold	564

### TEs in the coelacanth transcriptome

**Figure 5:**
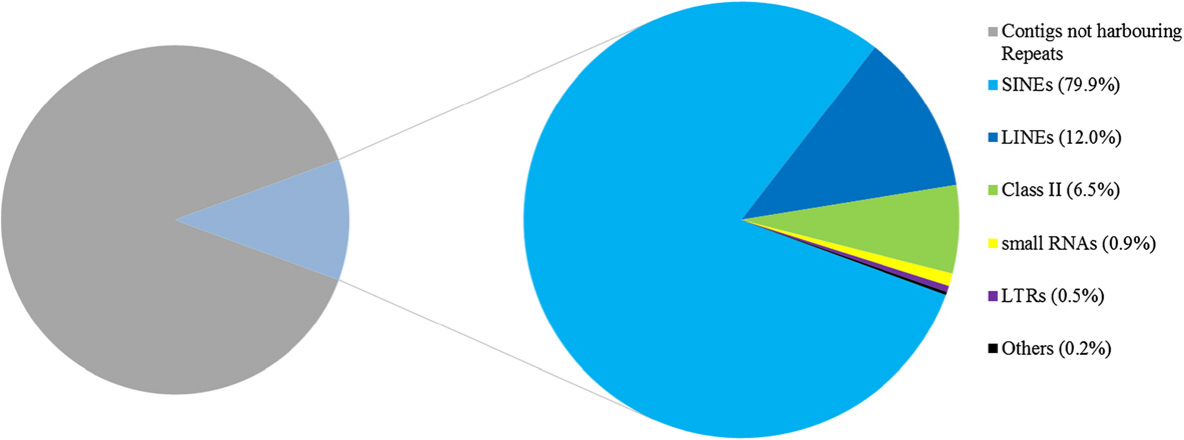
**Contigs harbouring a repeat element identified by RepeatMasker.** SINEs (Short interspersed elements); LINEs (Long interspersed elements); Class II (DNA transposons); small RNAs (non-coding RNAs: tRNAs, srpRNAs, snRNAs, 7SK RNAs); LTRs (Long terminal repeats); the “others” category comprises unknown and satellite elements.

The analysis carried out with RepeatMasker (http://www.repeatmasker.org) to identify transcribed repetitive elements of *L. menadoensis* revealed that 11.17% of the assembled contigs harbored at least one repeat and that 1.87% of sequenced bases matched to a RepeatMasker entry. The major part of matching contigs harbored a transposable element (98.9%): SINEs (79.9%), LINEs (12.0%), LTR retrotransposon families (0.5%), and DNA transposons (6.5%). The types of repetitive elements less represented were small RNAs such as tRNAs, srpRNAs, snRNAs, and 7SK RNAs (0.9%), and Unknown and Satellite elements (0.2%) (Figure 5). Furthermore the InterPro domain analysis on the 66,308 high quality contigs allowed to identify 119 transcripts containing the IPR000477 domain (Reverse transcriptase), 72 contigs with IPR004244 domain (Transposase, L1), and 17 sequences harboring IPR001584 domain (Integrase, catalytic core).

### RNA-seq mapping on the African coelacanth genome

Globally, the 61.64% of the trimmed sequencing reads of liver and testis RNA could map to the genes annotated of *L. chalumnae*. Ninety three percent of the counted fragments mapped within exons (51.63% were mapped on exon-exon junctions), whereas just 6.97% of the counted fragments mapped within introns (2.98% mapped on exon-intron junctions). The observed redundancy was very low, highlighted by a match specificity of 98.1% and 17,129 out of the 22,819 annotated gene models were found to have a positive mapping, meaning that the 75.06% of the coelacanth predicted genes were expressed in liver or in testis. A larger proportion of reads could map to the full assembled genome (85,682,920), revealing that 34.77% of the reads account for the expression of genes which are still not annotated. Overall, 3,189,494 reads (3.59% out of the total) could not be mapped to the *L. chalumnae* genomic scaffolds. The summary of the RNA-seq data mapping on the African coelacanth genome is presented in Table 4.

**Table 4:** **RNA-seq mapping on *L. chalumnae *genome statistics**

**Mapping on annotated genes**	
Counted fragments	21,589,809 (48.59%)
Uncounted fragments	22,846,398 (51.41%)
Match specificity	98.1%
Reads mapped in pairs	43,179,618 (48.59%)
Reads mapped in broken pairs	11,603,481 (13.06%)
Unmapped reads	34,089,315 (38.36%)
Fragments mapped on exon-exon junctions	11,147,648 (51.63%)
Fragments mapped on exon-intron junctions	642,299 (2.98%)
Total fragments mapped on exons	20,084,744 (93.03%)
Total fragments mapped on introns	1,505,065 (6.97%)
**Mapping on genomic scaffolds**	
Total number of reads mapping on genomic scaffolds	85,682,920 (96.41%)
Reads mapped on non-annotated genes	30,899,821 (34.77%)
Unmapped reads	3,189,494 (3.59%)

### RNA-seq mapping on *L. menadoensis* transcriptome

The RNA-seq mapping performed to calculate the expression levels of the assembled transcripts in both analyzed organs mapped the majority of paired-end reads (Table 5). In fact, the percentage of counted fragments was 67.20% in liver and slightly lower in testis, 64.57%, whereas the fraction of mapping reads was very similar in the two organs, being 78.12% in liver and 76.90% in testis, indicating that slightly more than 20% of reads could not be mapped to the final set of assembled contigs. Furthermore, it was possible to estimate the number of fragments which were not used at all by the assembly procedure, by comparing the number of paired-end reads mapping in broken pairs with the number of uncounted fragments. Only about 5.5% out of the total number of fragments produced by sequencing did not show any mapping, neither as intact nor as broken pairs (5.47% in liver and 5.38% in testis).

**Table 5:** RNA-seq statistics

**Liver**	**Liver**	**Testis**
Counted fragments	15,949,179	13,363,810
Percentage of counted fragments	67.20%	64.57%
-uniquely	13,479,204	10,734,166
-non-specifically	2,469,975	2,629,644
Uncounted fragments	7,786,110	7,337,108
Reads mapped in pairs	31,898,358	26,727,620
Reads mapped in broken pairs	5,187,130	5,111,602
Percentage of mapped reads	78.12%	76.9%
Reads not mapped	10,385,090	9,562,614

**Figure 6:**
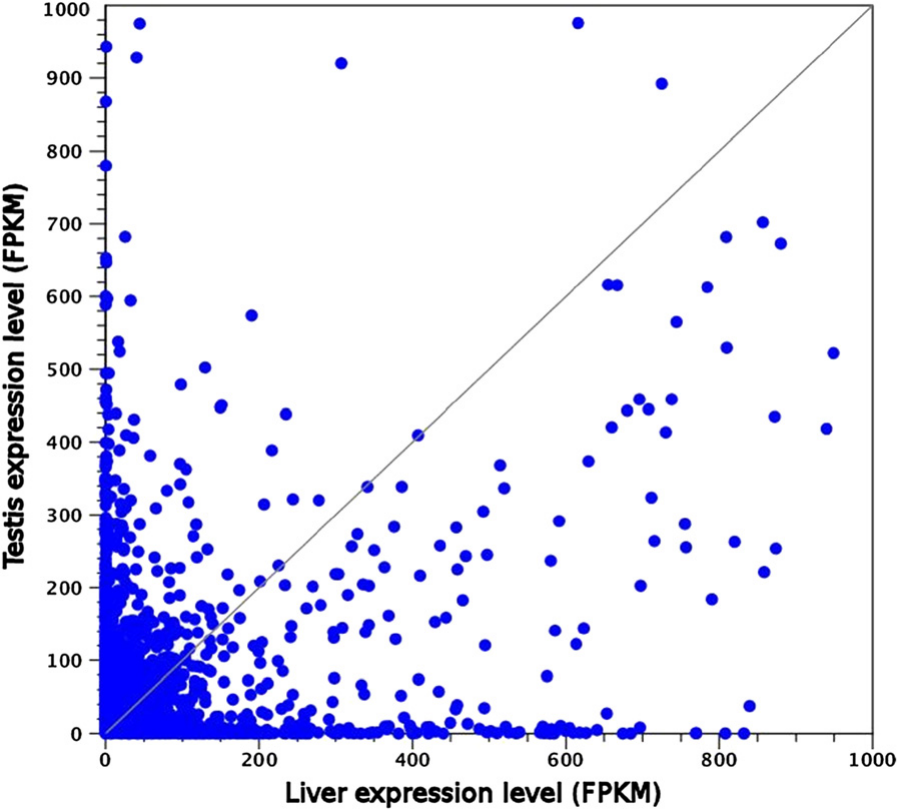
**Scatter plot depicting the expression levels (calculated as FPKM, Fragments Per Kilobase per Million fragments mapped) in liver and testis.** Genes whose expression levels are identical in the two organs are located on the bisector. For graphical representation convenience, only genes whose expression was lower than 1,000 FPKM in both tissues are shown (therefore 79 genes are not shown in the graph).

The RNA-seq mapping revealed that a higher number of transcripts were expressed in testis in respect to liver, since the expression of 55,975 contigs (84.42%) was found in liver, whereas the expression of 61,633 sequences (92.95%) was detected in testis. The comparison between the two organs highlighted that 51,302 contigs (77.37%) were expressed in both. Nevertheless, the two transcriptomes resulted to be remarkably divergent when comparing expression levels, which for most genes were largely divergent as shown by the expression scatter plot in Figure 6. The list of the 20 most expressed transcripts in liver and testis is reported in Tables 6 and 7, respectively. With a few exceptions (most notably the *elongation factor 1 α* and the *ATP synthase F0 subunit 6*, whose expression is important for the correct maintenance of all cell types) the 20 genes characterizing the two tissues show great differences in expression.

**Table 6:** List of the 20 most expressed genes in liver

**Gene name**	Liver expression level (FPKM*)	Testis expression level (FPKM*)
*α-2-macroglobulin like 1*	20,843	4
*Apolipoprotein AIV-like*	13,161	1
*Inner centromere protein A*	12,980	29
*Vitellin layer outer membrane 1*	11,015	1
*Fibrinogen α chain*	8,185	<1
*Fibrinogen β chain*	7,773	1
*Hemopexin*	7,131	1
*Elongation factor 1-α*	6,729	2,517
*Serine proteinase inhibitor Kazal type 2*	5,698	<1
*Ferritin H*	5,000	613
*ATP synthase F0 subunit 6*	4,648	1,680
*Lipocalin*	4,279	314
*Apolipoprotein E*	4,210	46
*Ferritin heavy polypeptide 1*	3,826	340
*Riboflavin-binding protein*	3,676	12
*Serum albumin*	3,601	<1
*α-2 macroglobulin*	3,548	<1
*Fibrinogen gamma polypeptide*	3,482	14
*Vitronectin*	3,481	<1
*Serum amyloid P*	3,345	15

*Fragments Per Kilobase per Million fragments mapped.

**Table 7:** List of the 20 most expressed genes in testis

**Gene name**	Testis expression level (FPKM*)	**Liver expression level (FPKM)**
*Testis-specific histone*	156,928	39
*Prostaglandin H2D isomerase*	6,494	5
*Y-box transcription factor*	4,264	359
*Sjogren syndrome nuclear autoantigen 1*	3,899	0
*Tubulin α chain, testis-specific*	3,318	26
*Elongation factor 1-α*	2,517	6,729
*Histone H1x-like*	2,033	7
*H\ACA ribonucleoprotein complex, subunit 2*	1,992	4
*Unknown*	1,953	1
*Tubulin β 2-C*	1,758	9
*ATP synthase F0 subunit 6*	1,680	4,648
*Sperm nuclear basic protein PL-I*	1,439	1
*Centrin-1*	1,346	5
*Ferritin heavy chain*	1,224	650
*HSP90-β*	1,214	508
*Ubiquitin*	1,212	425
*Cra-B*	1,152	1
*TP-53 target gene protein-like*	1,139	<1
*Ribosomal protein S6*	1,013	1,691
*High mobility group protein B2*	976	616

*Fragments Per Kilobase per Million fragments mapped.

**Figure 7:**
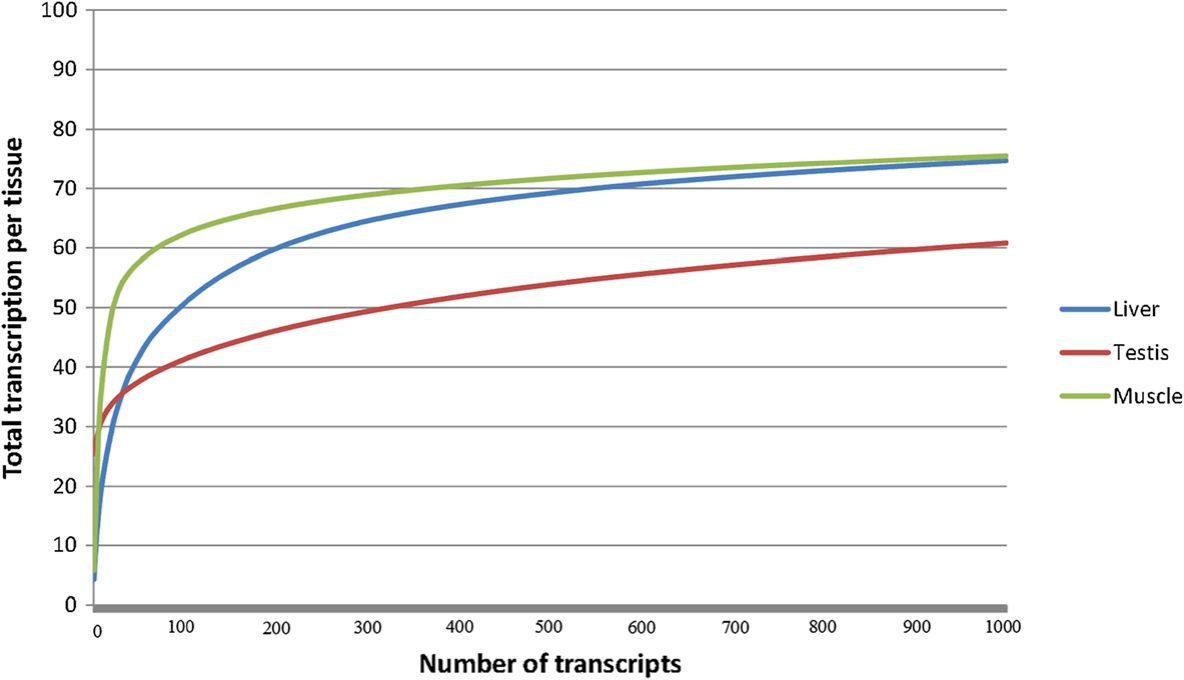
**Transcriptomic richness of *L. menadoensis *liver and testis and of *L. chalumnae *muscle.** Data are shown as the cumulative FPKM values of the 1,000 most expressed transcripts per each tissue, normalized on the total expression (y-axis). Modified from supplementary materials of Amemiya *et al*. [38].

The transcriptome richness was further graphically inspected in Figure 7 comparing *L. menadoensis* liver and testis transcriptomes to the RNA-seq of *L. chalumnae* muscle. A steep curve, that reaches quickly the asymptote (corresponding to the 100% of the transcription observed in each tissue), means that a low number of genes are expressed at high levels in a specific tissue. On the contrary, the later the curve approaches the asymptote, the more genes are expressed, indicating higher transcriptome richness. Among the 3 tissues, muscle is the one characterized by the steepest curve, as the 50% of the total gene expression in this organ is given by just 22 genes, consistently with observations previously collected in other organisms [44]. The two tissues used for the deep RNA-seq of *L. menadoensis* were both richer than muscle, although testis resulted to be, by far, the one expressing a broader range of transcripts. In this tissue 325 genes contributed to 50% of gene expression, while in liver the same number of genes accounted for about 65%. The 1,000 most expressed genes in liver and muscle contributed to about 75% of total transcription, whereas the same number of genes in testis just contributed to 61%.

**Figure 8:**
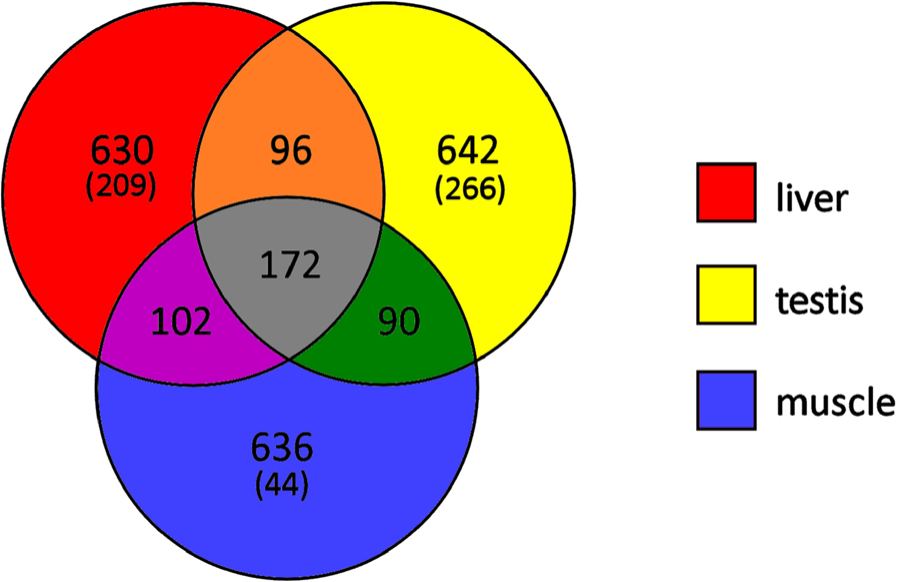
**Venn diagram depicting the overlap between liver, testis and muscle transcriptomes.** The overlap is evaluated on the 1,000 most expressed transcripts in each tissue (modified from supplementary materials of Amemiya *et al*. [38]). The values between brackets indicate the number of highly tissue-specific genes (found among the 1,000 most expressed in one tissue but displaying a FPKM value lower than 1 in the other two).

The overlap between liver, testis and muscle transcriptomes was further investigated by analyzing how many common genes were found among the 1,000 most expressed in each tissue. A schematic representation of transcriptomes overlap is given in the Venn diagram in Figure 8. 172 sequences, likely representing housekeeping genes, whose expression at rather elevated levels is important in all tissues, were found in all the 3 sets. In all the three organs analyzed, about 2/3 of the transcripts were identified as tissue specific, highlighting once again the strong link between the biological function of different tissues and gene expression.

## Discussion

### *De novo* transcriptome assembly

The advent of NGS technologies has had an outstanding impact on many fields of biology, including genetics [45], functional and comparative genomics [46,47] and molecular ecology [48]. The remarkable potential range of application of these techniques will likely move the focus of high throughput sequencing in the near future from genome and transcriptome sequencing to the use in clinical medicine and diagnostics [49-51]. Due to its potential application to deep RNA-seq, NGS has been praised as a cost-effective and revolutionary tool for transcriptomics since the very early stages of its development [52]. Although great technical advances have been made in a relatively short lapse of time in the improvement of both sequencing technologies and sequencing data management, significant challenges linked with RNA-seq still remain unsolved. The major computational issues in the management of NGS data is represented by the reliable *de novo* assembly of transcriptomes [53,54]. This is a complex task, due to presence of alternatively spliced transcript variants, gene duplications, allelic polymorphisms and noise due to suboptimal sequence quality, which often leads to the generation of a high number of short and poorly assembled contigs [55].

The massive amount of sequencing reads obtained from *L. menadoensis* liver and testis allowed us to apply stringent filtering criteria, both in the processing of raw sequencing reads and in the filtering of assembled contigs, in order to achieve a final set of high quality transcripts and to overcome the most common pitfalls of NGS assemblies. We chose to use the Trinity assembler, able to efficiently recover full length transcripts across a broad range of expression levels but somewhat redundant because of the inclusion of alternatively spliced variants [39]. The Trinity assembly was used as a reference sequence set to be appropriately refined and enriched, whenever possible, by a second *de novo* assembly performed with the assembler included in the CLC Genomic Workbench. The choice of integrating the Trinity output with the CLC assembly was made because of the empirical observation of a more effective reconstruction of full length transcripts and because of the operational speed of its assembly algorithm, based on de Bruijn graph. As this method, although extremely fast, is known to produce assemblies which are quite fragmented in comparison with other assemblers [56], only a selected set of assembled contigs was used to improve the Trinity assembly, with a particular emphasis on protein-coding transcripts.

### *De novo* assembly quality assessment

One of the problems most commonly arising from the *de novo* assembly of RNA-seq data is represented by sequence fragmentation [55]. In order to minimize this problem, as described in the methods section, all the contigs with an average coverage lower than 5 were removed prior to further analysis, reducing the number of contigs from 105,653 to a final set of 66,308 high quality sequences, reducing the fraction of short sequences with a proportional enrichment in longer transcripts (Figure 3). Furthermore, the contig processing strategy we used, graphically summarized in Figure 1, contributed to significantly reduce the sequence redundancy of the assembly (which was calculated to be 17.39%), in respect with the Trinity output (Figure 2). Although several factors can negatively influence the outcome of a *de novo* transcriptome assembly, affecting the reconstruction of full length sequences, the ortholog hit ratio analysis highlighted good mean and median ratio values and a high proportion of transcripts assembled to their full length (Additional file 1: Figure S1e). Therefore, despite the inevitable presence of broken transcripts, the results of the *de novo* assembly were extremely satisfying, highlighting that about half of the sequences, contained in the final set of transcripts, was assembled to the full length or very close to it and that just about a quarter of the contigs were resulting from highly fragmented transcripts.

### Transcript annotation

The analysis of the top hit species distribution resulting from BLAST (Figure 5) reveals *Gallus gallus* as the first species, followed by *Xenopus tropicalis*. The first teleost fish of the list, *Danio rerio*, ranked at the sixth place of the list, after the mammal *Monodelphis domestica*. These results are clearly biased towards organisms whose genome has been largely and deeply studied and annotated, mainly because of the higher quality of genome assemblies, of the more accurate gene predictions and of the higher number of protein sequences deposited in public sequence databases. Nevertheless, the absence of a prominent species with extended sequence homologies to *L. menadoensis*, neither in fishes nor in tetrapods, is consistent with the phylogenetic placement of lobe-finned fishes. However, for an in-depth analysis of the phylogenetic relationship between coelacanth and these two major vertebrate groups, and for an extended discussion on the implications on tetrapod evolution we refer to the whole-genome scale analysis reported by Amemiya and colleagues [38].

Compared to those having a positive BLAST result, a higher number of contigs (42,667) were annotated by InterProScan. Since the presence of InterPro domains is a strong indication of coding sequences, these data point out that 64.35% of the coelacanth *de novo* assembled contigs are coding for proteins characterized by known InterPro domains.

### Divergence between the two coelacanth species

The evolutionary divergence between the two species of coelacanth has been a subject of debate for a long time. Although the complete sequencing of mitochondrial DNA highlighted a sequence identity of 96%, variable divergence times have been proposed, ranging from 6 to 40 Mya [7,10]. The sequencing of the genome of *L. chalumnae* permitted to extend the comparison to large genomic regions with the available BACs of *L. menadoensis*, evidencing an identity of 98.7%. Our transcriptomic data offered the opportunity to assess the sequence identity within the coding regions, which resulted to be surprisingly high, standing at 99.73% (see Additional file 1 for methodological details). Nevertheless, while the massive amount of information gathered permits a rather easy calculation of divergence rates, the estimate of divergence time is not such a trivial task, given the uncertainties related to the calibration of a molecular clock [57]: the slow generation time, the absence of other closely related living species, and the allegedly low rate of molecular evolution of coelacanths.

Although both the genic and genomic divergence between the two species are similar to those observed between human and chimp [38], which diverged 6–8 Mya, we also performed a phylogenomic comparison between coelacanths and *T. rubripes*/*T. nigroviridis*, two organisms with a completely sequenced genome, which evolved in an aquatic environment and were subject to somewhat similar selective pressure and whose divergence, based on paleontological evidence, is estimated between 32.25 and 56 Mya [58]. Based on the alignment of approximately 40 Kb of ortholog transcribed sequences in *Latimeria*, we estimated the substitution rate to be 0,49/100 bp, whereas the substitution rate in the same set of selected genes in the *Takifugu*/*Tetraodon* pair was approximately 16 times higher (8,25/100 bp). A simple molecular clock correlation would indicate that the dating of divergence between African and Indonesian coelacanth should be placed between 1.9 and 3.3 Mya. Nevertheless the slower rate of molecular evolution, as well as the considerably longer generation time have to be taken into account, likely moving the divergence time much back in time to a date close to the lower end of the estimates based on mitochondrial DNA.

### TEs discussion

In metazoans repeat elements cover a considerable part of genomes. Moreover, the transcriptome analysis allowed the evaluation of the transcriptional activity of transposable elements (TEs) which play a key role in gene evolution and genome plasticity. TEs are divided in two classes: Class I is composed of Long Terminal Repeat retrotransposons (LTRs) and Non-LTRs (subdivided in LINEs and SINEs); Class II is composed of DNA transposons.

The RepeatMasker analysis revealed that 11.17% of contigs harbored a repeat and the most represented elements belong to SINE families. The latter result is in line with the studies performed in the Indonesian coelacanth genome [31-33], in which the activity of SINE elements in *Latimeria* was inferred. The identification of LF-SINEs and DeuSINEs in *L. menadoensis* transcriptome might confirm that these elements are actually active. Moreover, since their conservation in higher vertebrates, this movement might predate the common ancestor of Crossopterygians, for more than 400 Myr. On the other hand the occurrence of complete SINEs in contigs bearing protein-coding sequence might reveal the gain of new functional roles (exaptation) [59], as previously described in tetrapod genomes.

Concerning the activity of LINEs, the second most represented interspersed elements, the InterProScan analysis identified amino acidic domains linked to these autonomous retrotransposons. Chicken Repeat 1 (CR1) elements are the most abundant among LINEs. In contrast to the *G. gallus* genome where these elements are predominant but, with very few exceptions, nonfunctional [60], in *Latimeria* they seem to be active. Fragmented LTRs and ERVs (Endogenous RetroViruses) were identified in the transcriptome. This result is in agreement with the analyses on Foamy-like retroviral elements recently discovered in *L. chalumnae* genome by Han and Worobey [61] showing many frame-shifts and stop codons. The abundance of the *Harbinger* DNA transposons in *L. menadoensis* genome [34] suggests that Class II elements represent a remarkable fraction of the coelacanth TEs, however our analysis indicates that few DNA elements are expressed. This discordance may be related to the lack of coelacanth specific sequences belonging to this class in the RM database or to their propagation mode. The identification of mobile elements in transcriptomes sheds light on an unexpected genome dynamicity in an organism considered to be a living fossil even from a molecular point of view [21,25].

### RNA-seq mapping on the African coelacanth genome

More than half of the sequence data generated by the RNA-seq of *L. menadoensis* liver and testis mapped on the genes annotated by Ensembl on the *L. chalumnae* genome (Table 4), revealing an overall good annotation of the African coelacanth transcripts, even though in some cases the RNA-seq data produced in this study could provide some evidence of additional exons and alternative splicing, given that the 6.97% of the reads corresponded to regions annotated as introns.

Nevertheless, a rather high proportion of reads, close to 40%, could not be mapped on the genes annotated by Ensembl, consistently with the strategy adopted by Ensembl for the annotation pipeline, which is automated and mainly focused on protein-coding gene models. In fact, almost the 35% of the sequencing reads could map on the assembled genomic scaffolds outside from the annotated gene boundaries, revealing that a relevant portion of the transcripts expressed in the Indonesian coelacanth liver and testis might correspond to genes which were not annotated by the Ensembl RNA-seq annotation pipeline (Table 4). Therefore, the deep RNA-seq of liver and testis can be considered as a fundamental tool for the discovery of novel genes, and in particular, of many not yet annotated non-coding transcripts. As a matter of fact, the NGS sequence data will certainly provide a fundamental source of information for the study of atypical transcripts originated by trans- and circular splicing events, a topic which is currently under investigation (Stadler, personal communication).

Slightly more than 3 million reads did not map on the genomic scaffolds. These sequence data could either correspond to mitochondrial RNA (which was esteemed to account for 3.03% and 2.08% of the reads in liver and testis, respectively) or to coding genes harbored in *L. chalumnae* genomic regions which were not successfully assembled.

### Liver and testis transcriptomes comparison

The expression profile of the two organs analyzed was expected to be quite different, considering the largely different tasks they perform and the highly specialized cellular types involved. This difference was immediately evident by the graphical representation of the expression scatter plot (Figure 6). Among the 20 most expressed transcripts in liver, a large fraction is constituted by plasma proteins, whose synthesis is carried out by this organ (such as the three chains constituting fibrinogen, α-2 macroglobulins, apolipoproteins, hemopexin, vitronectin, lipocalin, serum amyloid P and serum albumin) and which constitute the core of the highly expressed genes in this tissue (Table 6). On the other hand testis invests a significant portion of transcription in genes involved in chromatin and cytoskeletal rearrangements. In particular, a testis-specific histone results to be expressed almost 25 times more than the second most expressed gene, *prostaglandin H2D-isomerase*, and accounts for about 18% of the global testis transcription. A significant amount of the total gene expression is derived from the synthesis of messengers of protamines, used for the replacement of histones and the effective packaging of DNA in the sperm acrosome [62]. The expression of genes involved in chromatin rearrangement is strictly regulated, as testis-specific histones are transiently and selectively expressed only during specific phases of spermiogenesis [63]. In fact, also *sperm nuclear basic protein PL-I* and *histone H1x-like* figure among the most representative testis genes. Furthermore a relevant number of other testis-specific genes can be linked to the meiotic process carried out in the testicular germinal cells and to the cytoskeletal rearrangements consequently required (*tubulin α chain testis-specific*, *tubulin β 2-C* and *centrin-1*). Moreover, specific types of microtubules are required for the correct assembly of mitotic and meiotic spindles and of the flagellum axoneme of spermatozoa [64,65]. The tubulin genes highly expressed in testis are likely linked to these functions.

Although the expression of a large fraction of genes was clearly strictly tissue-specific, thanks to the sequencing depth applied, a relevant overlap between the two transcriptomes (77.37%) was observed. The issue of transcriptome richness was addressed by analyzing the relative contributions of the expression of each contig to the total observed transcription in the two tissues, and in RNA-seq data of *L. chalumnae* muscle (Figure 7). Highly specialized tissues are expected to invest the most gene expression in a selected set of genes, thus being transcriptionally poor, whereas tissues involved in many different biological functions, characterized by the coexistence of many different cell types are expected to be transcriptionally rich, as they express a broader range of transcripts. Within this picture, muscle is a classic example of a highly specialized tissue, expressing at particularly high levels a limited set of genes involved in the structural organization of muscle fibers and responsible of contraction. Testis expresses a broader range of transcripts, which is in agreement with the assumption that cells in this organ are characterized by drastic morphological and functional changes linked to gamete generation: in this scenario chromatin structure is constantly rearranged and gene expression may therefore be substantially variable during the different stages of spermatogenesis [66]. Despite being transcriptionally poorer than testis, the RNA-seq of liver likely brought a remarkable amount of additional data as pointed out by the extent of the overlap between the two transcriptomes (Figure 8). Therefore, although the RNA-seq of two different organs like testis and liver was particularly effective to approach the coverage of a complete transcriptome, the incomplete overlap observed between the two *L. menadoensis* transcriptomes and the *L. chalumnae* muscle suggests that the sequencing of RNAs obtained from additional samples would be useful in order to effectively describe the complete transcriptome of this organism.

## Conclusions

The *de novo* assembly of the Indonesian coelacanth *L. menadoensis* liver and testis transcriptomes here described contains complete information concerning the expressed sequences involved in the important biological processes held by liver and testis tissues, such as metabolism and reproduction [30,67]. Furthermore, thanks to the high sequencing depth applied and to the broad range of transcripts expressed, the assembly also contains a great amount of sequence data originated from genes which are not directly linked to liver and testis, permitting to obtain a good overview of the overall coelacanth transcriptome.

In addition, the RNA-seq data generated in the present work provided a valuable resource for the Ensembl annotation of the recently sequenced genome of the African coelacanth *L. chalumnae.* In fact, the paired-end sequence data from liver and testis were processed through the Ensembl RNA-seq pipeline, generating 9,364 high confidence gene models, which permitted to improve the genome annotation by the addition of 547 new genes and 1,782 related transcripts [38]. This was a considerable improvement with respect to the previous annotation, based both on sequence similarity and on the data provided by the RNA-seq of *L. chalumnae* muscle, a tissue transcriptionally poor if compared with liver and testis.

Overall, the RNA-seq data here described represent a valuable source of information for the improvement of the genetic and genomic studies of coelacanths, including the analysis of TE activity, the occurrence of atypical splice events and the divergence between the African and Indonesian congeners. The importance of the obtained results mainly regards the origin of terrestrial vertebrates since the key position of *Latimeria* as the unique extant representative of the lineage from which tetrapods should have arisen. The transcriptome data indicate a higher affinity of this species to several terrestrial vertebrates, even if only in few species the genome and transcriptome have been exhaustively analyzed. Therefore, the *de novo* transcriptome assembly, for the quality of information it generated, may certainly be considered a step ahead in helping to understand the biology of this living fossil.

## Methods

### Samples collection

On 16th September 2009 a coelacanth was found in a shark net near Talise Island, Indonesia. This male, weighing 27 kg with a total length of 116 cm, was the fifth specimen of *L. menadoensis* ever caught since the discovery of this species in 1997. The animal was moved to the Faculty of Fisheries and Marine Science, University of Sam Ratulangi, Manado (Indonesia), where the liver and testis tissues used in this study were collected immediately after death and directly fixed in RNAlater (Applied Biosystems, Warrington, UK). Tissue samples were shipped to the Science Faculty, Università Politecnica delle Marche, Ancona, Italy, under the Convention on International Trade in Endangered Species (CITES; permit no. IT/IM/2009/MCE/01585-2009/19713).

### RNA extraction

Total RNA was isolated from liver and testis using TRIzol reagent (Invitrogen, Carlsbad, CA). Following the treatment with DNase I Amplification Grade (Sigma, Steinheim, Germany), an aliquot of the extracts was used to assess the quality and quantity of RNA by spectrophotometric and capillary electrophoretic analysis. The liver RNA sample resulted to have a 260/280 nm absorbance ratio of 1.74, a 260/230 nm absorbance ratio of 0.94 and a RNA integrity number (RIN, estimated with an Agilent2100 Bioanalyzer) of 6.6. The testis RNA sample resulted to have a 260/280 and a 260/230 nm absorbance ratios of 1.89 and 1.23, respectively, with a RIN of 7.

### Sequencing of the liver and testis transcriptomes

Messenger RNA selection and cDNA library preparation were performed by the Istituto di Genomica Applicata (IGA, Udine, Italy). The sequencing of the libraries was performed on an Illumina Genome Analyzer II platform (San Diego, California). Briefly, the poly-A mRNAs were selected using magnetic beads-linked oligo (dT) probes. The fragmentation was obtained with divalent cations. cDNA was synthetized and Illumina sequencing adapters were then ligated to the fragments, according to the manufacturer’s protocol. A smear of ligated fragments of 150 to 400 bp of length was selected by size and excised from an agarose gel. The sequencing of the cDNA libraries was performed on a flow cell using a 100-cycles paired-end strategy.

### Data processing and *de novo* assembly of *Latimeria menadoensis* transcriptome

The raw sequencing reads were trimmed by removing Illumina adapter sequences and low quality bases (the quality limit was set to 0.05). The resulting trimmed sequences shorter than 75 bp were discarded. All the reads originated from ribosomal RNA were also removed prior to the assembly step.

The *de novo* assembly of the processed reads was performed with a combined approach, by integrating the outputs of two different methods, which have been specifically developed for *de novo* assembly of short reads: Trinity [39] and the commercially available CLC Genomic Workbench 4.5.1 (CLC Bio, Katrinebjerg, Denmark). At first, the two assemblies were performed separately using as input the same sequence set, comprising both liver and testis sequence data. The schematic summary of the procedure used for integrating the outputs of the two assemblers is detailed in Additional file 1.

To ensure the creation of a highly reliable set of assembled transcripts, contigs covered by a low number of reads were discarded, following a global mapping of the complete set of both liver and testis filtered reads (CLC Genomic Workbench, mismatch/insertion/deletion costs set at 2/3/3, length fraction/similarity set to 0.75/0.95). All the transcripts showing an average coverage <5 were considered as possible fragments of longer transcripts, not reliable enough to be included in the high quality coelacanth transcript collection, and were therefore discarded. Only transcripts longer than 249 bp were kept.

### Assembly quality assessment

In order to assess the quality of the contigs obtained with the filtering procedure in respect with the non-filtered set, the sequences were grouped into categories according to their sizes (intervals of 100 bp) and the relative abundance of each category was plotted in a histogram. The distributions of transcript lengths pre- and post-filtering were compared.

The sequence redundancy was estimated by the RNA-seq mapping of the reads from both tissues on the contigs created by the original Trinity assembly and to the filtered and non-filtered sets of contigs obtained with the Trinity and CLC combined approach. The RNA-seq analysis tool included in the CLC Genomic Workbench was used for this purpose (minimum length fraction and minimum similarity fraction were set at 0.75 and 0.95, respectively). The total number of reads mapped and the set of reads mapping non-specifically (matching on more than 1 contig) were compared, in order to evaluate the improvement of the assembly quality obtained with the processing steps. Sequence redundancy was calculated as the percentage of reads mapping not-specifically.

The total number of reads originated from mitochondrial RNA was assessed by the mapping of the filtered reads set to the deposited mitochondrial DNA sequence of *L. menadoensis* (Genebank accession: NC_006921.2). The mapping was performed with the CLC Genomic Workbench, using the same settings described above to estimate sequence redundancy.

### Transcript functional annotation

The filtered transcripts were annotated with Blast2GO (version 2.4.4, http://www.blast2go.com/), a tool specifically developed for the annotation of novel sequence sets [68]. Sequence similarity was evaluated with BLASTx [69] against the NCBI non-redundant (nr) protein database using an e-value cutoff of 1×10^-6^. The presence of conserved domains was researched and annotated using InterProScan [70] on the six possible translation frames of each contig. Contigs were functionally annotated according to the Gene Ontology (http://www.geneontology.org/) nomenclature. GO terms were assigned to each transcript and annotated according to the level 2 of the Cell Component, Molecular Function, and Biological Process categories. Furthermore, in order to identify by homology transposable elements and repeated sequences from a database of vertebrate repeats, the contigs were analyzed with RepeatMasker (http://www.repeatmasker.org).

### Mapping on *L. chalumnae* genome

The liver and testis sets of filtered reads were mapped on the annotated *L. chalumnae* genome Ensembl release e!67 using the Genomic Workbench 4.5.1 RNA-seq tool, assuming a minimum length fraction of 0.75 and a minimum similarity fraction allowed of 0.95. As the sequence similarity between *L. menadoensis* and *L. chalumnae* was estimated to be 99.73%, the mapping parameters used were supposed not to significantly influence the mapping outcome. The allowed paired-end read distance was set between 100 and 350 bp. Based on gene annotations, it was possible to categorize the fragments as mapping within exons, within introns and on exon-exon or exon-intron junctions. Furthermore, the number of reads mapping on non-annotated genomic regions was also calculated, to assess the amount of sequence data accounting for the expression of non-annotated genes.

### RNA-seq analysis

The liver and testis filtered reads were separately mapped to the high quality set of the assembled contigs to assess the expression values in the two tissues. The mapping was carried out with the Genomic Workbench 4.5.1 RNA-seq tool, with a minimum length fraction allowed of 0.75 and a minimum similarity fraction allowed of 0.95. Paired-end read distance was considered to be comprised between 100 and 350 bp. Only intact sequence pairs (fragments) mapping were counted and expression values were calculated as FPKM (Fragments Per Kilobase per Million fragments mapped). Besides liver and testis, also RNA-seq data obtained from the African congener *L. chalumnae* muscle (Sequence Read Archive sample ID: SRS283232) were used for comparison purpose (the muscle transcriptome was *de novo* assembled with Trinity and processed to remove redundancy exactly as previously described for the liver and testis assembly).

The transcriptome richness was graphically inspected by plotting the cumulative expression of the 1,000 most transcribed genes in each tissue, normalized on the total expression per tissue. The overlap between liver and testis transcriptomes was estimated by the comparison of the sets comprising the 1,000 most expressed genes per tissue. The comparison was also extended to the *L. chalumnae* muscle transcript set generated in the frame of the African coelacanth genome sequencing project [38].

### Availability of supporting data

[SRA: SRS362269, SRA: SRS362270]

## Competing interests

The authors declare that they have no competing interests.

## Authors’ contributions

EO, AC, AP and GS planned and coordinated the project. DMM collected the tissue samples. MF, MAB, MB, AC and EO prepared and sequenced the liver and testis RNA-seq libraries and performed the transposable elements analysis. AP, MG, GDM and JT-M performed the transcriptome assembly and annotation. AP, MG, GDM, AC, MF, MAB, MB, EO, GS, FB and AMF performed the transcriptome analyses. JA, FDP and JT-M compared the transcriptome data with the African coelacanth genome. AP, AC, MG, GDM and MF wrote the paper with input from other authors. All authors read and approved the final manuscript.

## Supplementary Material

Additional file 1: Figure S1 **a**: Distribution of average sequence quality scores. The quality score for each read is calculated as the arithmetic mean of its base qualities. PHRED score is represented on the x-axis, the proportion of sequences observed at each score is shown on the y-axis. **b**: Coverage for the four nucleotides and ambiguous bases. The base position relative to each read is indicated on the x-axis, the percentage of each nucleotide observed at a certain position is shown on the y-axis. **c**: Combined coverage of G and C bases. The base position is shown on the x-axis, the percentage of G and C bases observed at each position is shown on the y-axis. **d**: Combined coverage of ambiguous bases. The base position is shown on the x-axis, the percentage of ambiguous bases observed at each position is shown on the y-axis. **e**: Ortholog Hit Ratio, calculated on the high quality set of liver and testis transcripts. The ratio of length between assembled contigs and the full length orthologs is reported on the x-axis, the percentage of contigs observed in each ratio category is shown on the y-axis. **f**: Gene Ontology mapping performed on the high quality transcript set. The mapping summary takes into account annotations at Level 2 of Cell Component, Molecular Function and Biological Process. **Supplementary Methods:** transcriptome assembly; transcripts integrity evaluation; comparison between the two coelacanth species. Click here for file
