# Supplementary material for: Analysis of the transcriptome of the Indonesian coelacanth Latimeria menadoensis
Source: BMC Genomics. 2013 Aug 8;14:538. doi: 10.1186/1471-2164-14-538 (PMC3750513; doi:10.1186/1471-2164-14-538)
Supplement: Additional file 1: Figure S1 — a: Distribution of average sequence quality scores. The quality score for each read is calculated as the arithmetic mean of its base qualities. PHRED score is represented on the x-axis, the proportion of sequences observed at each score is shown on the y-axis. b: Coverage for the four nucleotides and ambiguous bases. The base position relative to each read is indicated on the x-axis, the percentage of each nucleotide observed at a certain position is shown on the y-axis. c: Combined coverage of G and C bases. The base position is shown on the x-axis, the percentage of G and C bases observed at each position is shown on the y-axis. d: Combined coverage of ambiguous bases. The base position is shown on the x-axis, the percentage of ambiguous bases observed at each position is shown on the y-axis. e: Ortholog Hit Ratio, calculated on the high quality set of liver and testis transcripts. The ratio of length between assembled contigs and the full length orthologs is reported on the x-axis, the percentage of contigs observed in each ratio category is shown on the y-axis. f: Gene Ontology mapping performed on the high quality transcript set. The mapping summary takes into account annotations at Level 2 of Cell Component, Molecular Function and Biological Process. Supplementary Methods: transcriptome assembly; transcripts integrity evaluation; comparison between the two coelacanth species. [file 1471-2164-14-538-S1.docx]

**BMC Genomics**

**Analysis of the transcriptome of the Indonesian coelacanth: *Latimeria menadoensis*.**

Alberto Pallavicini, Adriana Canapa, Marco Barucca, Jessica Alföldi, Maria Assunta Biscotti, Francesco Buonocore, Gianluca De Moro, Federica Di Palma, Anna Maria Fausto, Mariko Forconi, Marco Gerdol, Daisy Monica Makapedua, Ettore Olmo, Jason Turner-Meier_,_ Giuseppe Scapigliati

**Additional File 1**


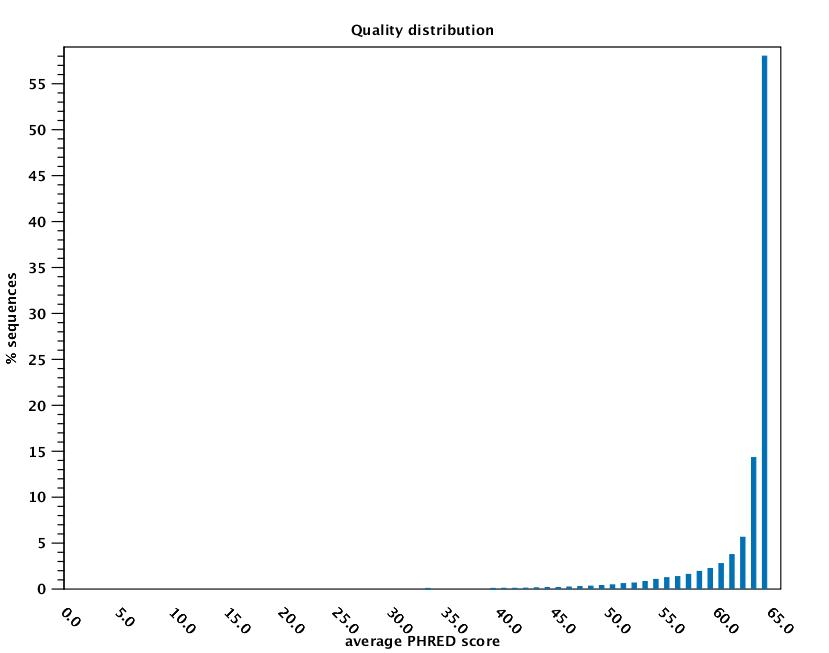


**Figure S1a:** Distribution of average sequence quality scores. The quality score for each read is calculated as the arithmetic mean of its base qualities. PHRED score is represented on the x-axis, the proportion of sequences observed at each score is shown on the y-axis.


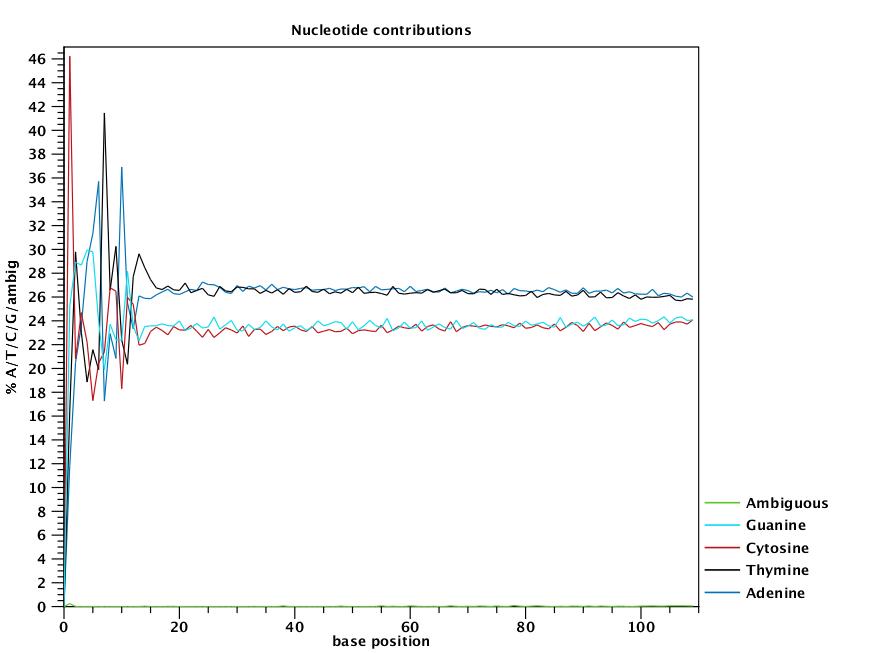


**Figure S1b:** Coverage for the four nucleotides and ambiguous bases. The base position relative to each read is indicated on the x-axis, the percentage of each nucleotide observed at a certain position is shown on the y-axis.


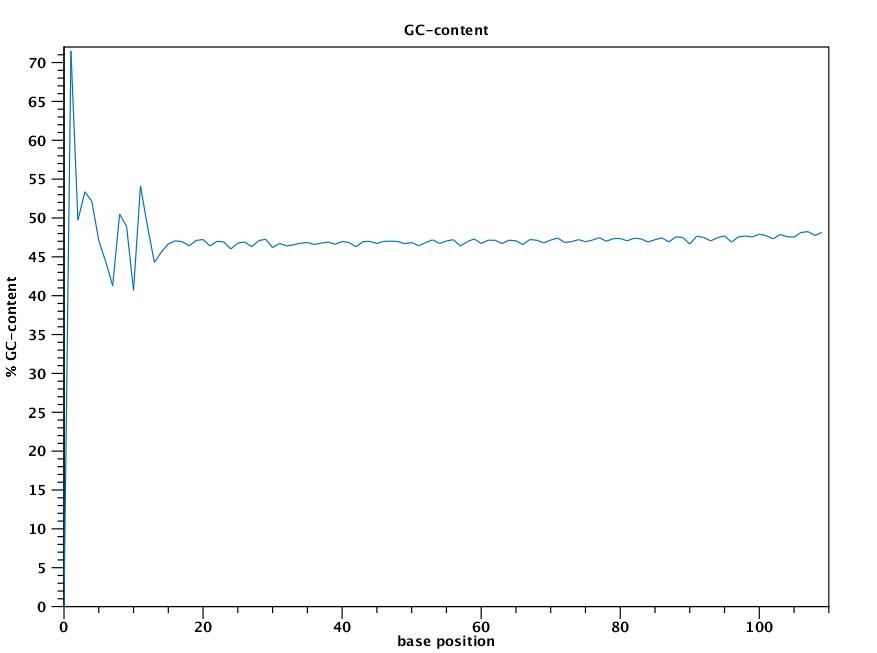


**Figure S1c:** Combined coverage of G and C bases. The base position is shown on the x-axis, the percentage of G and C bases observed at each position is shown on the y-axis.


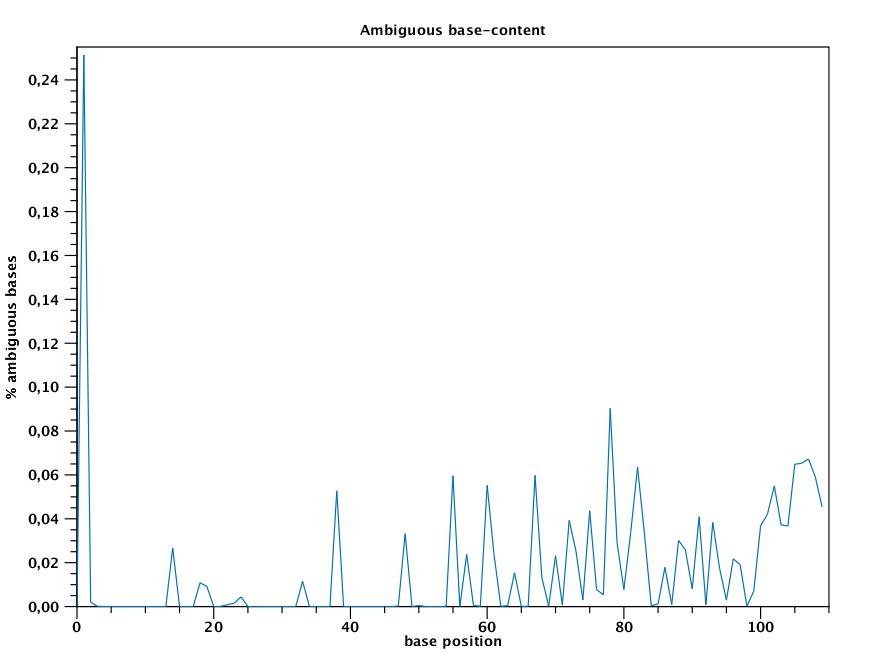


**Figure S1d:** Combined coverage of ambiguous bases. The base position is shown on the x-axis, the percentage of ambiguous bases observed at each position is shown on the y-axis.


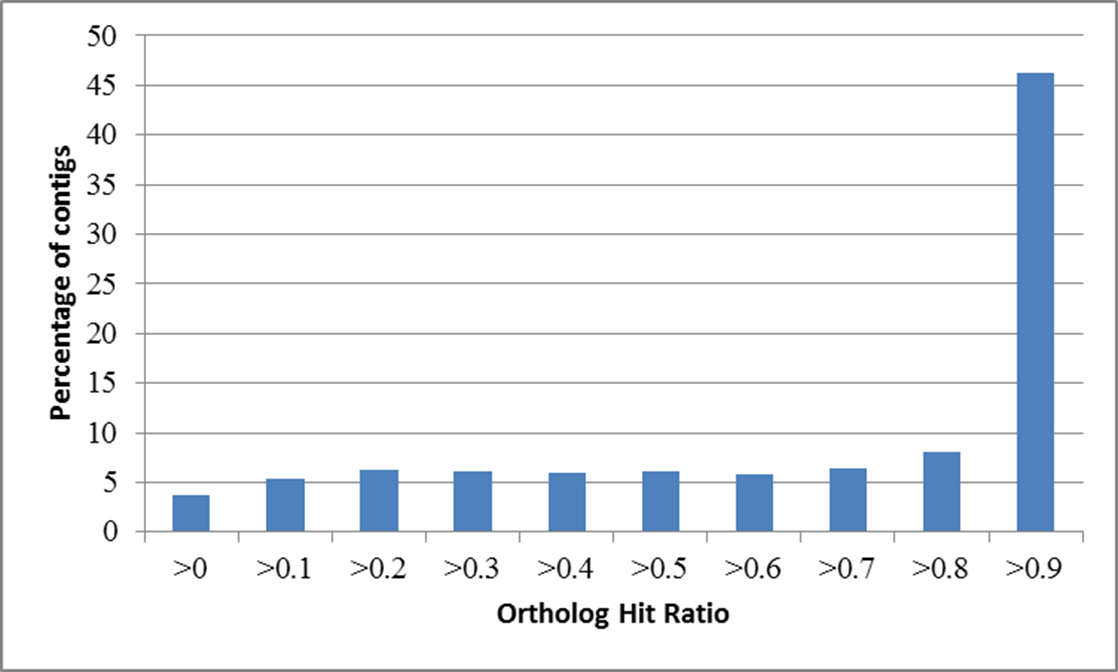


**Figure S1e**: Ortholog Hit Ratio, calculated on the high quality set of liver and testis transcripts. The ratio of length between assembled contigs and the full length orthologs is reported on the x-axis, the percentage of contigs observed in each ratio category is shown on the y-axis.


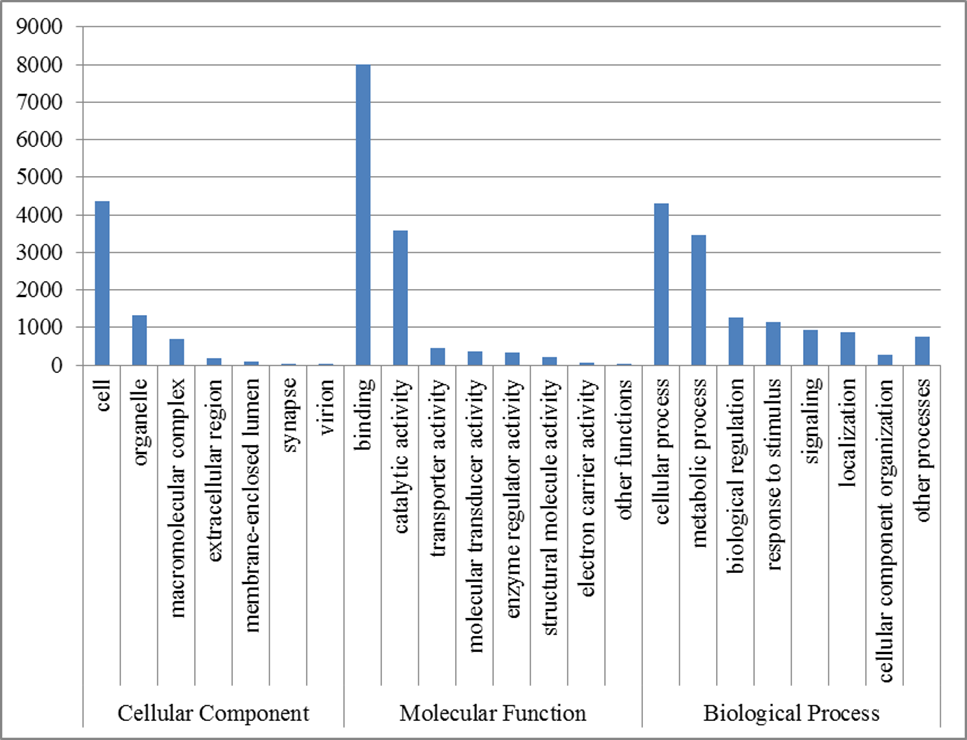


**Figure S1f**: Gene Ontology mapping performed on the high quality transcript set. The mapping summary takes into account annotations at Level 2 of Cell Component, Molecular Function and Biological Process.

**Supplementary Methods**

**Transcriptome assembly**

The de novo Trinity assembly was completed using the November 2011 version of Trinity. It was run using the strand-specific data option which was set to RF. All other options were set to their default values. Only the longest transcripts per each gene were selected for further analysis. Redundant and overlapping contigs created by Trinity were collapsed by a MIRA 3.4.0 assembly [1].

The *de novo* CLC assembly was performed assuming a paired-end read distance comprised between 100 and 350 bp and the penalties for mismatches, insertions, and deletions were set at 2\3\3, whereas the parameters for the length fraction and similarity were set to 0.5 and 0.9, respectively. The paired-end read distance was empirically determined after several preliminary *de novo* assemblies followed by analysis of paired-end read mapping, which showed this range to be normally distributed with the highest frequency at 240 bp. The minimum allowed assembled contig length was set at 250 bp. Only contigs assembled with high confidence were kept and used for the implementation of the Trinity assembly whenever possible. A particular emphasis was put on protein-coding transcripts, as only contigs displaying an open reading frame (ORF) of a minimum of 70 codons were selected. The ORF prediction was carried out with the “Find Open Reading Frames” tool included in the CLC Genomic Workbench, considering AUG as a start codon and selecting the “open-ended sequence” option.

Identical or highly similar contigs generated by the two different *de novo* assemblers were detected by BLASTn, setting the cutoff to an e-value of 1x10^-100^ and to an identity of 98%. Contigs generated by the CLC assembler identical to those created by Trinity were discarded, unless they were extending the Trinity contigs by at least 200 bp. In the latter case, Trinity contigs were replaced by their CLC counterparts.

**Transcripts integrity evaluation**

The approximate abundance of full length transcripts and the fragmentation in the collection were estimated using the Ortholog Hit Ratio method [2], using the NCBI non-redundant (nr) protein database for the determination of the hit length regions through BLASTx. A correction was applied to the standard method in order to remove the bias given by inter-species divergence, as only contigs displaying BLASTx identity higher than 90%, independently from the alignment length, were considered as “true orthologs and selected for the analysis.

**Comparison between the two coelacanth species**

The identity percentage on a nucleotide level between *Latimeria chalumnae* and *Latimeria menadoensis* was calculated based on 5,608 coding sequences with a minimum length of 500 codons. Alignments were only considered within coding regions, detected with the “Find Open Reading Frames” tool included in CLC Genomic Workbench v5.1 (CLC Bio, Katrinebjerg, Germany) from the initial ATG to the final STOP codon, selecting the “open-ended sequence” option. Alignments were performed by BLASTn and only hits displaying an e-value lower than 1x10^-25^ and longer than 80 base pairs were considered.

For the comparative analysis between *L. chalumnae*/*L. menadoensis* and *T. rubripes*/*T. nigroviridis*, a set of 25 highly conserved, single copy, ortholog genes was selected. Only sequences available for all the 4 organisms (sharing a minimum identity of 80% on a nucleotide level between coelacanth and pufferfish by BLASTn) were used, overall accounting for approximately 40Kb of alignable sequence data.

**REFERENCES**

1. Chevreux B, Pfisterer T, Drescher B, Driesel AJ, Müller WEG, Wetter T, Suhai S: **Using the miraEST Assembler for Reliable and Automated mRNA Transcript Assembly and SNP Detection in Sequenced ESTs.** *Genome Research* 2004, **14**:1147-1159.

2. O'Neil S, Dzurisin J, Carmichael R, Lobo N, Emrich S, Hellmann J: P**opulation-level transcriptome sequencing of nonmodel organisms *Erynnis propertius* and *Papilio zelicaon*.** *BMC Genomics* 2010, **11**:310.
